# Social Representations of Hero and Everyday Hero: A Network Study from Representative Samples

**DOI:** 10.1371/journal.pone.0159354

**Published:** 2016-08-15

**Authors:** Zsolt Keczer, Bálint File, Gábor Orosz, Philip G. Zimbardo

**Affiliations:** 1 Eötvös Loránd University, Faculty of Education and Psychology, Institute of Psychology, Budapest, Hungary; 2 MTA Research Centre for Natural Sciences, Institute of Cognitive Neuroscience and Psychology, Budapest, Hungary; 3 Pázmány Péter Catholic University, Faculty of Information Technology and Bionics, Budapest, Hungary; 4 University of Szeged, Institute of Psychology, Szeged, Hungary; 5 Stanford University, Department of Psychology, Stanford, CA, United States of America; Koc Universitesi, TURKEY

## Abstract

The psychological investigation of heroism is relatively new. At this stage, inductive methods can shed light on its main aspects. Therefore, we examined the social representations of Hero and Everyday Hero by collecting word associations from two separate representative samples in Hungary. We constructed two networks from these word associations. The results show that the social representation of Hero is more centralized and it cannot be divided into smaller units. The network of Everyday Hero is divided into five units and the significance moves from abstract hero characteristics to concrete social roles and occupations exhibiting pro-social values. We also created networks from the common associations of Hero and Everyday Hero. The structures of these networks show a moderate similarity and the connections are more balanced in case of Everyday Hero. While heroism in general can be the source of inspiration, the promotion of everyday heroism can be more successful in encouraging ordinary people to recognize their own potential for heroic behavior.

## Introduction

Social psychology has relatively few empirical studies on heroism [1,2,3,4,5,6,7]. Recently, Kinsella et al. [4] have applied versatile methodologies on comprehensive samples to converge heroism into a scientific concept. To broaden previous analyses on heroism, we apply network theory. This approach is capable of finding patterns in the connections among elements in social representations.

The present study maps the social representations of Hero and Everyday Hero in Hungary by representing them as networks constructed from free associations. We identify modules of the networks and categorize the associations based on their topological positions in the association networks. In order to do that, we define global hubs as the most dominant associations of the whole social representation and modular hubs as the characteristic associations in the different modules. After assessing the two social representations, we analyze the overlapping set of associations.

We emphasize that our aim is not to rigorously define concepts like Hero and Everyday Hero (for such work see Kinsella et al. [4]). What we are after is to observe how the perception of heroism changes when we make this distinction. In order to do that, we combine three theoretical perspectives: heroism as a social construct, social representation theory and network theory. In the following three subsections, we provide a short overview of these approaches.

### Heroism

As we investigate heroism inductively, we concentrate on studies with a similar approach [2,4,5,6]. Goethals and Allison [2] constructed the “Great Eight” of heroism based on bottom-up categorization by independent coders. Sullivan and Venter [6] presented a list of hero characteristics sorted by the frequencies of occurrence in survey results. Rankin and Eagly [5] provided a list containing components of hero definitions gathered from study participants’ responses. Kinsella et al. [3,4] explored the social representation of heroism combining multiple methodologies, such as open-ended surveys, reaction-time tasks, surprise recall tasks and ratings scales. They also constructed the hero functions framework which consists of three hero functions: enhancement, moral modeling and protection [3].

Inductive studies are exposed to several biases. The concept of heroism is shaped by larger cultural and historical contexts as well [1]. Furthermore, different social groups can have different heroes even in the same culture [6]. In addition to the cultural and social relativity, Sullivan and Venter [6,7] showed evidence that study participants relate differently to heroes identified by themselves than to heroes identified by others, which highlights the functional significance of the “my hero” concept instead of heroes in general. Kinsella et al. [4] collected several hero-related concepts that are often merged with heroic narrative in the general discourse: leaders, role models, sport stars and celebrities. Franco, Blau and Zimbardo [1] also argued that the meaning of heroism might be overloaded with political and media influences. [1,2].

Farley [8] suggested a distinction between Big H Heroism and Small h Heroism on a theoretical basis. Big H heroism refers to outstanding acts that display prototypical heroism. They are possible only in rare circumstances and they require a high level of moral character or competence. Therefore, they are only accessible to a few people. Farley [8] suggested categories of Big H Heroism, such as Situational Heroism (once in a lifetime situations), Life-Long Heroism (constant effort in social issues) and Professional/“911” (as part of the job/career/duty). Small h Heroism refers to small but challenging good deeds. Small h Heroism does not necessarily imply grand or exceptional moral character or abilities. It usually happens in everyday circumstances and goes unnoticed by the public. Thus, the possibility of experiencing such situations is much higher. Farley’s distinction has already been applied in research but only in terms of Big H Heroism [9]. Other researchers created comprehensive taxonomies on heroism empirically [1,2].

Recently, social psychologists have highlighted the everyday aspects of heroism. Goethals and Allison [2] created a taxonomy based on the hero’s social-influence that resulted in 10 types of heroes. One type is called Transparent Hero. These heroes are everyday heroes such as nurses, teachers, fire fighters and first respondents in emergency situations. Their achievements often remain unnoticed. Staats et al. [10] defined heroism by a set of traits that are measurable in everyday phenomena. In their study, the students who scored higher on personality measures related to heroic traits such as bravery, honesty and empathy also reported less intention to cheat in tests. This behavior benefits the community by accepting the rules and challenges of honest competition. Therefore, they considered these students as academic heroes. Davis, Brunette, Allison and Stone [11] also promoted a heroic narrative to help students overcome academic challenges. Zimbardo [12] has popularized the concept of everyday heroes. The educational program of the Heroic Imagination Project (http://heroicimagination.org) trains ordinary people to perform extraordinary acts in challenging situations.

### Social representations

Social representations are ideas, opinions and attitudes shared by a social group regarding a social object [13,14]. Inductive social representation studies frequently apply free associations [15,16]. Moscovici [13,14] identified the figurative core of a social representation, based on which Abric [15,17,18,19] developed the central core vs. periphery hypothesis.

The central core of a social representation consists of only a few and relatively abstract associations and has a pervasively influential role by defining the meaning of the whole social representation. The central core has three main functions, namely generating the meaning of the representation, influencing connections between other less important associations and stabilizing the representation under altering environments. Furthermore, the central core provides relevant norms, behavioral action plans and stereotypes in certain situations. Two representations differ if their central cores contain different associations.

In contrast with the central core, periphery associations constitute the largest part of the representation. Their meanings are relatively concrete. The periphery operates as an interface between the environment and the central core. The periphery is responsible for the concretization of the representation and gradual changes of the social representation start on the periphery.

### Networks

Networks are used for exploring underlying relations in various datasets (e.g., innovation processes [20], metabolic relations [21], brain functional interactions [22]). Every network consists of a set of objects, in which some pairs of objects are connected to each other. The objects are called nodes and the link between two nodes is called an edge. A network is undirected if the edges represent symmetric relations between the nodes. A network is weighted if values are assigned to the edges. The weight or even the existence of an edge between two nodes is determined by a predefined logical system [22,23]. The node strength is the sum of weights attached to the edges of a given node.

Steyvers and Tenenebaum [24] showed that large semantic and association networks are scale-free. Scale-free networks have a small number of hubs (we refer to these hubs as global hubs). Hubs are nodes with outstanding number of edges in the network. Hubs are often defined based on an arbitrarily chosen threshold value considering the order of magnitude of node strengths in the given network [25,26,27,28]. The rest of the nodes are peripheral nodes with significantly lower number of edges.

Many real world networks can be divided into modules. Modules are subunits of the system with much denser connectivity within themselves than between other regions of the network. The elements constituting a given module probably share similar properties regarding the analyzed phenomenon. [25,29,30]. Palla et al. [31] provided an example for a modular network of word associations starting from the word “bright”. The network was divided into four modules: Intelligence, Astronomy, Light, and Colors. The word “bright” was connected to all of them but the modules revealed alternative meanings.

## Material and Methods

### Participants

This research employed two nationally representative probability samples of 506 (in case of Hero) and 503 (in case of Everyday Hero) Hungarians aged between 15 and 75 years. The participants were selected randomly from an internet-enabled panel including 15,000 members with the help of a research market company in March 2014. For the preparation of the sample, a multiple-step, proportionally stratified, probabilistic sampling method was employed.

Members of this panel used the Internet at least once a week. The panel demography is permanently filtered. More specifically, individuals are removed from the panel if they give responses too quickly (i.e., without paying attention to their response,) and/or have fake (or not used) e-mail addresses. The sample is nationally representative in terms of gender, age, level of education, and type of residence for those Hungarians who use the Internet at least once a week.

The final samples comprised N_H_ = 502 and N_EH_ = 502 respondents who gave valid answers (M_H_ = 239, F_H_ = 263; M_EH_ = 238, F_EH_ = 264) aged between 15 and 75 years (M_Hage_ = 44.4 years; SD_Hage_ = 16.2 years; M_EHage_ = 44.0 years; SD_EHage_ = 16.2 years). Regarding the highest completed level of education, 22.9%/23.1% (Hero/Everyday Hero) of the respondents had primary level of education, 24.9%/24.9% had vocational school degree, 31.5%/30.7% graduated from high school and 20.1%/21.3% had higher education degree. Regarding the place of residence, 18.9%/18.7% of the respondents lived in the capital city, 19.5%/18.5% lived in the county capitals, 31.7%/32.2% lived in towns and 29.9%/30.6% lived in villages.

### Measures

The Research Ethics Committee of the Faculty of Education and Psychology of Eötvös Loránd University approved this study. All participants provided their written informed consent to participate in this study through a check-box on the online platform. In case of underage participants, parents (passive consent) were informed about the topic of the research. The ethics committee approved this consent procedure. Respondents volunteered for the study and they did not receive compensation for the participation. Furthermore, they were assured of their anonymity. Data was collected via an online questionnaire. Participants were informed about the content of the questionnaire (e.g., Hero, Everyday Hero).

We used an associative task based on Abric’s [15,19] theoretical underpinnings and on Vergès’ [32] methodological (data gathering) assumptions. A respondent had to associate five words or expressions to one of the cues resulting in an individual representation. The cues were Hero or Everyday Hero. They can be differentiated on the following dimensions: range, publicity, challenge, prevalence and accessibility. These are described in Table 1.

**Table 1:** Differentiation between Hero and Everyday Hero.

Dimension	Hero	Everyday Hero	Literature
**Range**	Hero has an effect on a large number of people (one event with transforming effect on society and inspiring others).	Everyday Hero has a local/limited magnitude of effect.	Franco et al. [1]; Goethals and Allison [2]
**Publicity**	Hero gains more publicity and overlaps with celebrities, role models, sport stars and leaders.	Everyday Hero is unnoticed by the public.	Goethals and Allison [2]; Kinsella et al. [4]; Spears et al. [33]
**Challenge**	Hero faces significant social sacrifice or life-threatening risk.	Everyday Hero faces social challenge.	Becker and Eagly [34]; Franco et al. [1]; Johnson [35]; Lyons [36]; Staats et al. [10]
**Prevalence**	Heroic acts occur only rarely in special circumstances that one might never encounter in everyday situations.	Everyday heroism is frequently possible in everyday situations (e.g., courageous conversation or reporting cheating).	Lyons [36]; Johnson [35]; Elinder and Erikxon [37]; Franco, Blau and Zimbardo [1]; Staats et al. [10]
**Accessibility**	Hero is associated with special abilities or extraordinary character.	Everyday Hero is not associated with special personality traits or outstanding abilities.	Goethals and Allison [2], Smith et al. [9], Staats et al. [10], Ulhmann et al. [38], Williams [39], Zimbardo [12]

The instruction was: “Please, write 5 words which first come into your mind about Hero/Everyday hero. Evaluate them on the following scale: negative, neutral, positive”. The associations were not categorized. We followed Flament and Rouquette’s [40] lemmatization criteria.

### Setting up the networks

We algorithmically set up two networks which stand for the social representations of Hero and Everyday Hero in Hungary. To create such networks, we had to determine the nodes and the edges. We listed the different associations from the total set of associations to a given cue. The nodes represented these different associations. There was an edge between two associations if they were mentioned together by at least one study participant. The weight of an edge between two associations was equal to the number of times they were mentioned together. Therefore, the construction of networks was only directed by the co-occurrences of associations in the individual representations. More sophisticated methods are also available besides this relatively simple procedure. For example, it is possible to consider the rank order of the associations for each participant [15,18]. In the present case, edge weights based on rank order would result in an arbitrary effect on our networks.

The method is similar to item-based recommendation algorithms [41], in which an item (product, movie, book, etc.) is recommended to a user based on the general pattern of other users’ preferences. When a user buys an item, the algorithm recommends other products that were purchased by previous users who were also interested in the same item [42]. Therefore, products frequently purchased together are strongly linked and often recommended, while weakly tied items are not. In our networks, the associations played the role of products.

The construction of the networks can be summarized in three steps:

1. Participants who mentioned the same association at least twice were deleted.
2. We determined the nodes. We ignored associations which occurred only once. According to Abric [15,18] they belong to the far periphery and are not necessarily stable parts of the social representation. However, these elements constitute the major part of the representation. From a network perspective, these associations typically have only one connection, thus removing these links ensures a higher robustness of the network. These sparsely connected nodes can easily result in disconnected subnetworks which make the modular analysis more difficult. The removal of these nodes has no effect on the scale-free properties of the networks.
3. We determined the edge weight between every pair of associations, which was equal to the number of times the two associations were mentioned together. A strong edge between two associations meant that they were frequently mentioned together in the individual representations, while the absence of an edge referred to the complete separation of the two associations on the individual level.

The above-described process was applied to the Hero and Everyday Hero associations separately, thereby resulting in two weighted and undirected networks. After removing subjects mentioning the same association more than once, the number of subjects was 474 in case of Hero and 481 in case of Everyday Hero. After removing associations that occurred only once, the number of different associations was 222 in case of Hero and 210 in case of Everyday Hero. The total number of associations was 2006 in case of Hero and 1899 in the case of Everyday Hero. Further analyses (calculating scale-free properties, calculating modularity, finding global and modular hubs) were carried out on these reduced datasets.

We constructed common association networks for the two social representations. In this case, the nodes are the associations present in both the Hero and Everyday Hero networks. The edges and the edge weights are determined with the same method as in case of the social representation networks. Therefore, the common association networks are subnetworks extracted from the social representation networks.

### Scale-free topology and modularity

The scale-free topology of a network refers to the power-law function that the probability distribution function (*P*(*x*)) of the node strength (*x*) follows:

$$P(x)\sim x^{-\alpha },$$

where *α* is the scaling parameter [43]. The scaling parameter typically lies in the range 2 < *α* < 3 [44].

The power-law distribution of the normalized node strengths were tested separately for the Hero and Everyday Hero networks. The Maximum Likelihood Estimation fitting model determined the scaling parameter (*α*) of the power-law function and the minimum node strength (*X*_min_) for which the power law holds. For statistical comparison, datasets were generated with the same parameters (*X*_min_ and *α*) as the empirical datasets. According to the null hypothesis of the Kolmogorov-Smirnoff test, the generated dataset has the same distribution as the empirical dataset. Following Clauset et al. [44] we determined the significance level as .1. This means that we considered our networks scale-free if p>.1 (for the applied toolbox and a more detailed description see: http://tuvalu.santafe.edu/~aaronc/powerlaws/ [44]).

We investigated the modular organization of the association networks. In order to do that, smaller subnetworks (modules) were decomposed from the entire networks and the modularity value (*Q*) was calculated. It is given by the following formula:

$$\text{Q}=\sum_{\text{s}=1}^{\text{N}}\left[\frac{{\text{k}}_{\text{s}}}{\text{L}}-{\left(\frac{{\text{d}}_{\text{s}}}{2\text{L}}\right)}^{2}\right].$$

In this formula, *N* is the number of modules, *L* is the total sum of edge weights in the network, *k*_*s*_ is the sum of edge weights in module *s*, and *d*_*s*_ is the sum of the node strengths (the sum of edge weights belonging to a certain node) in module *s* [45]. A modular structure of a network with a high value of *Q* must comprise many within-module links and as few as possible between-module links. The Louvain algorithm [46] with fine-tuning [47] was applied to identify the modular partition with the highest possible modularity. The resulting modular structure can change run by run [46]. Therefore, we applied the algorithm for 10,000 independent iterations and we chose the partition with the highest modularity value.

We examined the hierarchical relationship between the resulting modules by applying a hierarchical agglomerative clustering technique. Two clusters are merged in each iteration based on the maximal modularity criteria between the i^th^ and (i-1)^th^ community structure of the network (for details see [45]). The construction of the complete dendogram can mark the cohesive modules of the social representation even if the difference between the modularity values of the i^th^ and (i-1)^th^ partitions is negative.

Degree-, weight-, and strength-preserving randomization [48] was applied to generate 4999 independent null models (random networks) for the social representations of both Hero and Everyday Hero. The modular organizations of the two social representation networks were tested by comparing their maximal modularity values to the corresponding random networks. We applied a nonparametric statistics (one-sided) to test whether the modularity value of the social representation networks differed from that of the random networks (for detailed description see: [49]). The significance level was defined strictly, which means we rejected the null hypothesis if the social representation network’s modularity value was always higher than the corresponding random networks’ modularity value.

### Normalization of node strengths

Normalized node strengths and normalized intramodular node strengths [25] were calculated. These characterize the importance of each node in the whole network and within its module, respectively. The normalized node strength of node *i* is determined as:

$$Normalized\;Node\;Strength_{i}=\frac{K_{i}}{\bar{K}},$$

where *K*_*i*_ is the node strength of node *i*, $\bar{K}$ is the average node strength in the network. Nodes with normalized node strength > 2.5 were classified as global hubs of the network.

The normalized intramodular node strength of node *i* is:

$$Normalized\;Intramodular\;Node\;Strength_{i}=\frac{K_{i}}{{\bar{K}}_{S_{i}}},$$

where *K*_*i*_ is the intramodular node strength of node *i* (sum of all edge weights between node *i* and all the other nodes in its own module, *S*), $\bar{K_{S_{i}}}$ is the average intramodular node strengths of all nodes in the module. Nodes with normalized intramodular node strength > 2.5 were classified as modular hubs of the network.

The network construction and analysis were carried out in Matlab 7.9.1 software. All of the applied network parameters are available at https://sites.google.com/site/bctnet/. ForceAtlas2 layout algorithm [50] (Implemented in Gephi 0.8.2) was used for visualizing the networks.

## Results

The number of negative associations in both social representations was negligible. It was 100 out of 2510 in case of Hero and 81 out of 2510 in case of Everyday Hero. Most of them occurred only once and thus they were removed from the networks. We ignored the valences of the remaining associations.

**Fig 1:**
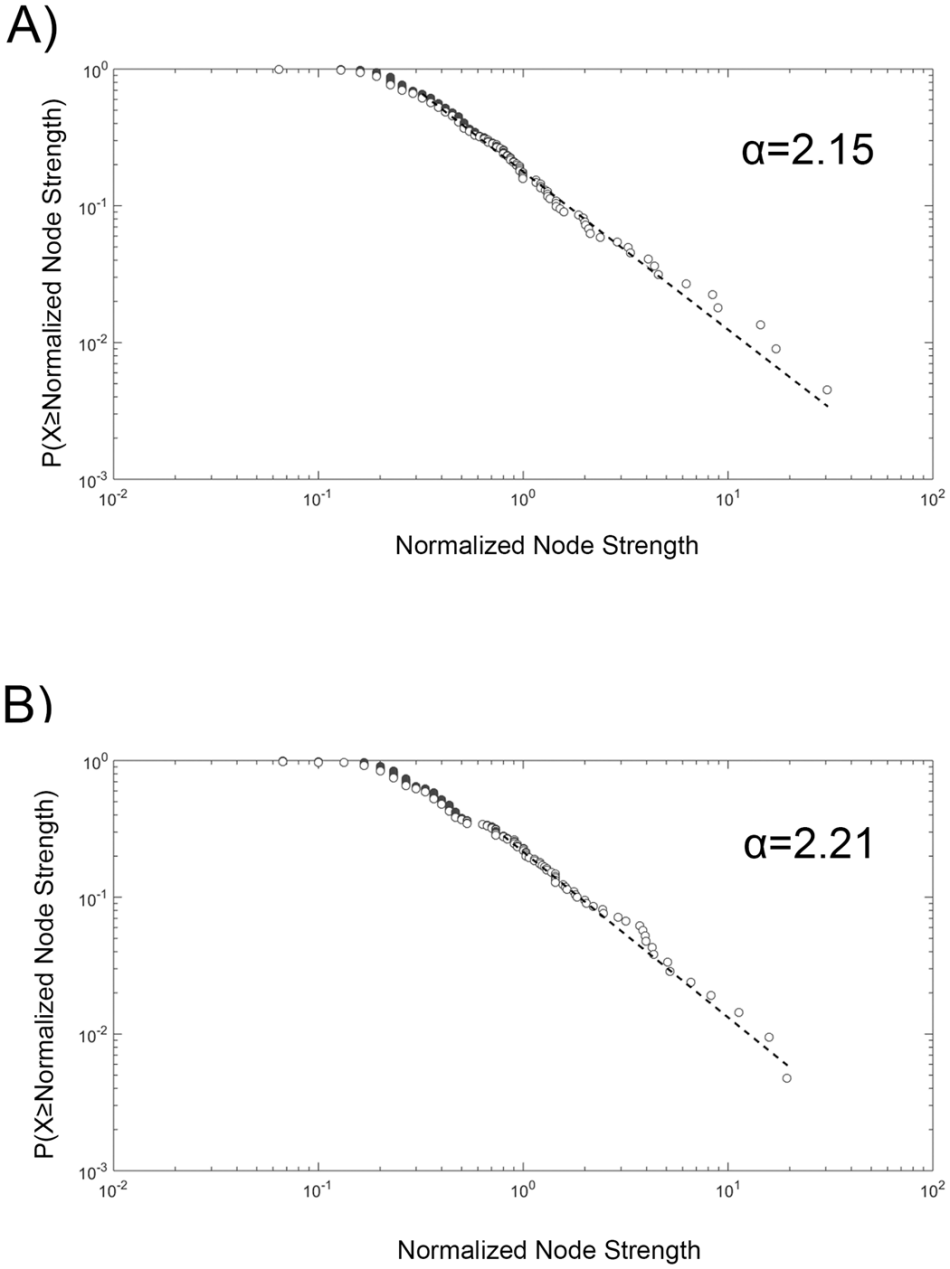
Scale-free properties of the Hero (A) and Everyday Hero (B) networks. The plots show the cumulative distribution functions of the normalized node strengths on log-log scales. The dashed, straight lines represent the Maximum Likelihood Estimation fitting of the data points. The power law exponents (*α*) for the Hero and Everyday Hero networks are 2.15 and 2.21, respectively.

Scale-free properties (scaling parameter (*α*), minimal normalized node strength (*X*_min_), p-value of the line fitting) were determined for the Hero and Everyday Hero networks. In case of Hero, we found *α* = 2.15 from *X*_min_ = .312. In case of Everyday Hero, we found *α* = 2.21 from *X*_min_ = .8. In the range determined by *X*_min_, the normalized node strength distributions showed a power law distribution (p(Hero) = .11, p(Everyday Hero) = .5). The log-log plots of the scale-free properties can be seen in Fig 1.

**Fig 2:**
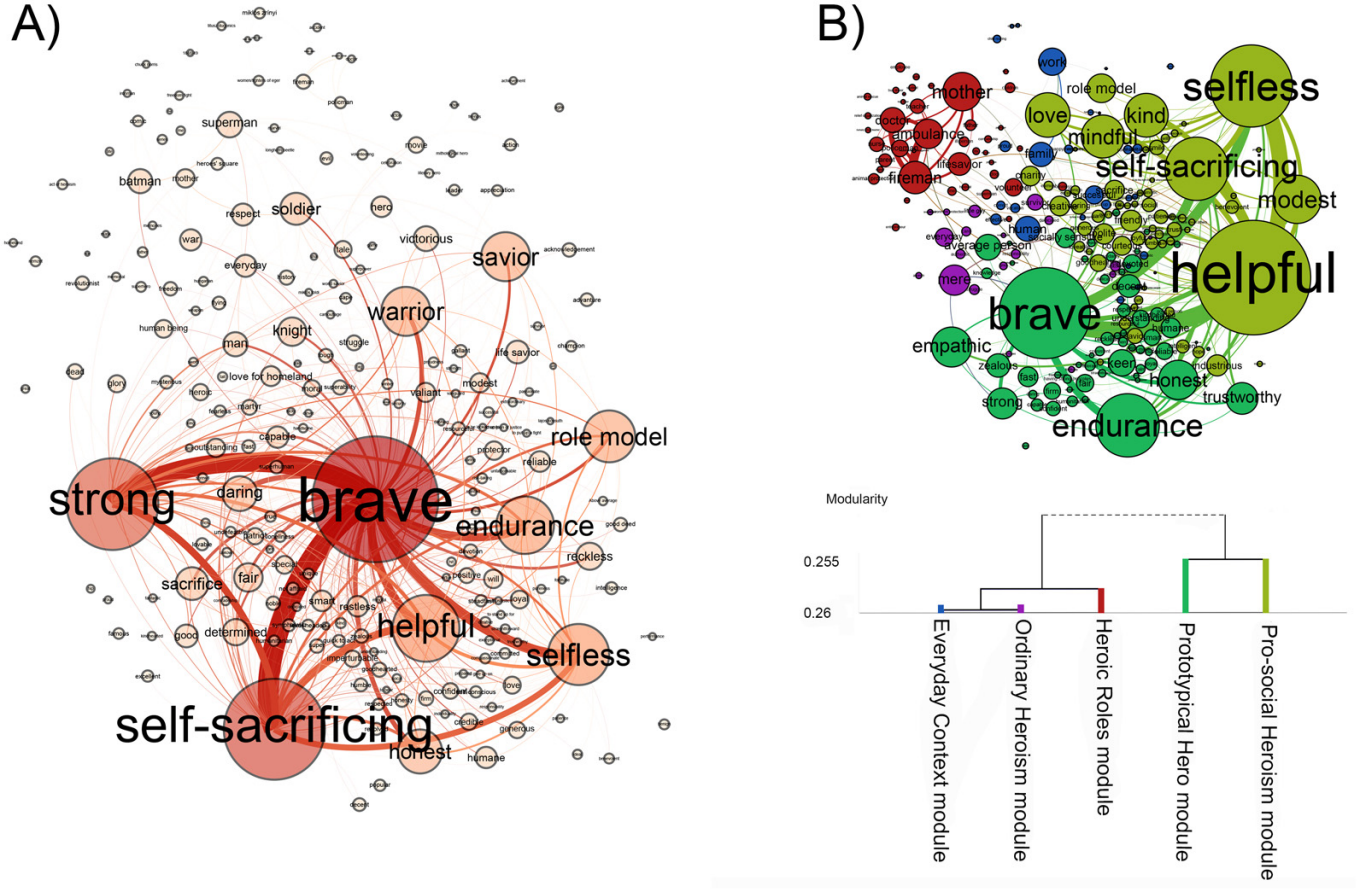
The social representations of Hero (A) and Everyday Hero (B). The association networks are visualized with the ForceAtlas 2 layout [50]. The size of a node denotes the node strength and the thickness of an edge refers to the edge weight. The networks were thresholded (edges below the value of 1 were deleted) for a better visualization. In case of Everyday Hero, nodes with the same color belong to the same module. The hierarchy and descriptive labels of the modules are presented on the dendrogram.

The modularity value of the Hero network (*Q* = .19) was not significantly higher than the corresponding modularity values of the null (random) models (p = .19; mean = .17; standard deviation = .027). In case of Everyday Hero, the modularity value of every (4999) independent null model was lower (p < .001; mean(random) = .15; standard deviation(random) = .013) than the modularity value calculated for the social representation network (*Q* = .26). These results showed that the Hero network was non-modular and the Everyday Hero network was modular. The visualization of the networks can be seen in Fig 2

We identified the global hubs of the Hero and Everyday Hero networks (Table 2). Hero is a network in which “brave” has an outstanding number of connections and it is followed by a couple of weaker global hubs. The global hubs of Hero are predominantly abstract values (“brave”, “self-sacrificing”, “strong”, “helpful”, “selfless”, “endurance”, “honest”, “daring” and “sacrifice”). Among the global hubs of Hero, three concrete nodes appear: “warrior”, “role-model” and “savior”. Everyday Hero also has both abstract and concrete global hubs. The concrete global hubs (“fireman”, “ambulance man”, “mother” and “doctor”) are roles and occupations associated with heroism. The abstract global hubs (“helpful”, “brave”, “selfless”, “self-sacrificing”, “endurance”, “modest”, “modest”, “honest”, “mindful”, “love”, “kind” and “emphatic”) are associations expressing heroic values.

**Table 2:** Global hubs of the Hero and Everyday Hero networks.

Hero	Normalized node strength>2.5	Everyday Hero	Normalized node strength>2.5
brave	30.45	Helpful	19.32
self-sacrificing	17.15	Brave	15.89
strong	14.42	Selfless	11.28
helpful	8.90	self-sacrificing	8.28
selfless	8.38	Endurance	6.61
endurance	6.26	Fireman	5.21
warrior	4.56	Modest	5.07
role model	4.37	Honest	4.34
honest	4.08	ambulance man	4.27
daring	3.34	Mindful	3.97
savior	3.24	Love	3.94
sacrifice	2.89	Kind	3.84
		Mother	3.70
		Doctor	3.17
		Empathic	2.90

Normalized node strength > 2.5 refers to the global hubs of the association network. The rest of the nodes and their normalized node strengths are available for Hero in S1 File and for Everyday Hero in S2 File.

In case of Everyday Hero, we identified five modules. We labeled each of them based on their modular hubs resulting in the following: Prototypical Hero module, Everyday Context module, Pro-social Heroism module, Ordinary Heroism module and Heroic Roles module (Table 3). Prototypical Hero and Pro-social Heroism belong to a superordinate group while Everyday Context, Ordinary Heroism, and Heroic Roles form another group (see the dendogram in Fig 2). Ordinary Heroism is a homogenous subnetwork and its nodes are relatively weakly tied. The only association that has node strength close to the threshold is “mere”.

**Table 3:** Modular hubs of Everyday Hero network.

Prototypical Hero	Everyday Context	Pro-social Heroism	Ordinary Heroism	Heroic Roles
Modular hubs (Normalized node strength>2.5)	Modular hubs (Normalized node strength>2.5)	Modular hubs (Normalized node strength>2.5)	Modular hubs (Normalized node strength>2.5)*	Modular hubs (Normalized node strength>2.5)
brave	work	Helpful		fireman
endurance	successful	Selfless		ambulance man
honest	family	self-sacrificing		doctor
strong		Modest		mother
		Kind		policeman
		Mindful		
		Love		

Normalized node strength > 2.5 refers to the modular hubs of the association network. The rest of the nodes and their normalized intra-modular node strengths are available in S3 File.

*All nodes of the module are below the threshold value.

We calculated how many concrete social roles and contexts are present in the social representation of Hero. We found that 38 out the 222 nodes (17.1%) were occupations (e.g., doctor, fireman, etc.), social roles (e.g., warrior, savior, etc.) or concrete characters (e.g., superheroes, historical figures, etc.).

### Structural differences of the common associations

**Fig 3:**
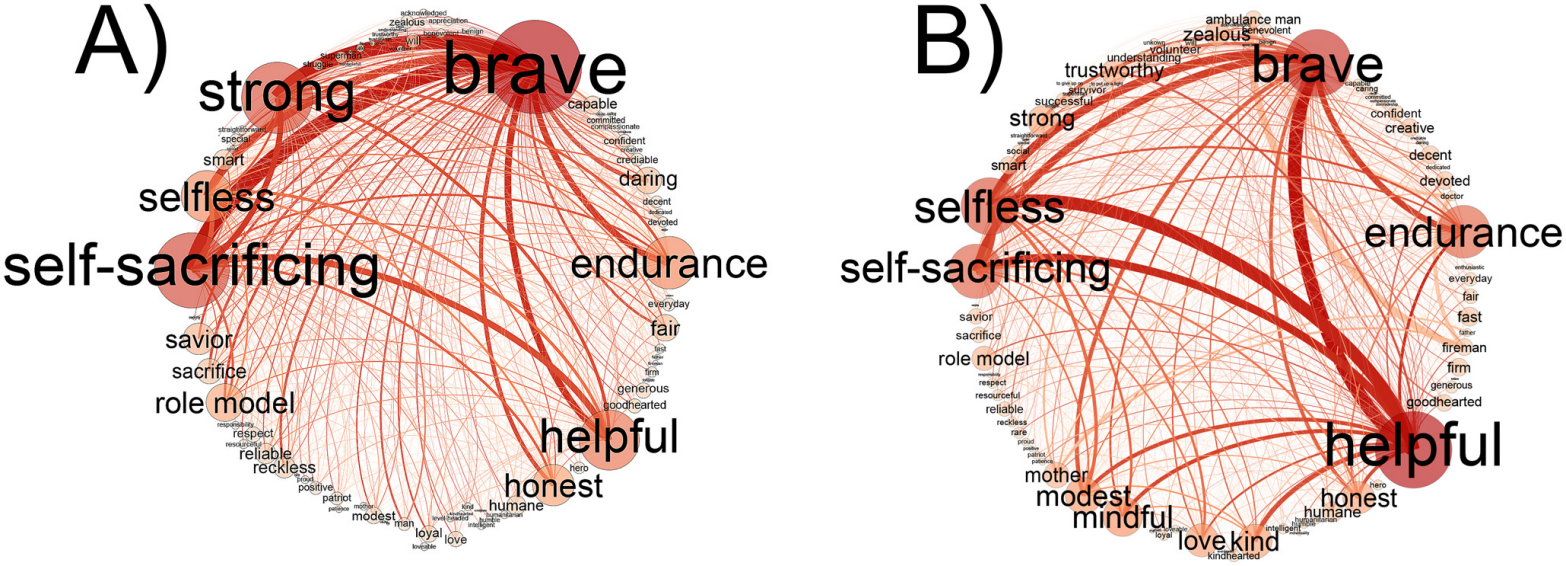
Common associations in the social representations of Hero (A) and Everyday Hero (B). The associations are arranged in a circular alphabetic order. The size of a node denotes the node strength and the thickness of an edge refers to the edge weight. The network was thresholded (edges below the value of 1 were deleted) for a better visualization.

We gathered the common associations of the two social representations and created two networks from them representing either Hero or Everyday Hero (Fig 3). The number of common associations was 85. The list of common associations is available in S4 File. A moderate correlation (r < .58, p < .001) was determined for the edge weights connecting the same nodes in the common association networks. In case of Hero, the majority of the edges are connected to “brave” with dominant links to “strong” and “self-sacrificing” (see in Fig 3A). In case of Everyday Hero, the dominant edges are more balanced between “helpful”, “selfless”, “self-sacrificing” and “brave” and even the less important edges seem to be more homogenously distributed (see in Fig 3B). All modules of Everyday Hero were present among the common associations (the quotient of the number of participating nodes from a module and all nodes of the module expressed in percentage) as follows: Prototypical Hero module: 55%; Everyday Context module: 32%; Pro-social Heroism module: 40%, Ordinary Heroism module: 40%; Heroic Roles module: 26%. The global hubs of the original networks were among the nodes of the common association networks except for “warrior” in Hero and “empathic” in Everyday Hero. Prototypical Hero module, Pro-social Heroism module and Ordinary Heroism module overlap to the highest degree with the social representation of Hero. These modules contain abstract heroic values and characteristics. Everyday Context module and Heroic Roles module are present in lower proportion among common associations. They are more concrete in terms of content. They contain social roles, occupations and social contexts.

## Discussion

Both social representations consist of only positive associations, which means that both Hero and Everyday Hero generate positive first impressions. The uniformity of valences also supports our assumption that a cue activates one cognitive schema and the first five associations are derived from it.

In case of both networks, we identified their global hubs. We applied Abric’s [15,18] central core vs. periphery approach as a theoretical framework which is consistent with our knowledge of hubs in scale-free networks [43,51]. Beyond the network interpretation of the classical Abric [18] model (central core of the social representation = the set of global hubs), we determined the modules and their modular hubs, which represent socio-cognitive patterns in the social representations based on Wachelke’s theoretical assumptions [52].

### Global hubs in the Hero and Everyday Hero networks

In case of Hero, our findings are in accordance with central and peripheral features provided by Kinsella et al. [4]. The social representation of Hero can be summarized as a collection of abstract values and characteristics. Although certain situations lead us to develop a heroic narrative [2,53], our findings imply that it is difficult to determine what exactly makes someone a hero. This is present in the lack of well-defined boundaries with other related terms, such as celebrities, sport stars, role models and martyrs [1,4,53,54]. The three concrete elements, “warrior”, “role model” and “savior” fit into the heroic prototype of several studies (altruism plus risk, rescuers) [35,36,55] but they are still not well-defined social roles or occupations. These concrete nodes can be associated with Farley’s [8] Situational Heroism and Goethals and Allison’s [2] Traditional Hero type. They can be also interpreted in terms of Franco et al.’s [1] physical risk-taking categories.

In the present data, the social representations of Hero and Everyday Hero cannot be divided in the same manner as Farley separated Small h and Big H Heroism [8]. The social representation of Everyday Hero shares some of the prototypical heroic characteristics (“brave”, “self-sacrificing”, “endurance”) with Hero, which relates it to Big H Heroism. Even its concrete elements can be associated with subcategories of Big H Heroism [8] such as Life-Long Heroism (“mother”) and Professional/”911” Heroism (“fireman”, “ambulance man”, “doctor”). However, the emphasis moves from the Big H Heroism theme (“brave”, “self-sacrificing”, “endurance”) to the Small h Heroism theme (“helpful”, “selfless”, “modest”, “mindful”, “love”, “kind”).

Both social representations have global hubs that refer to accessibility and prevalence. The global hubs of Hero express less specific expectations, which stems from the more abstract contents. Among the global hubs of Everyday Hero, there are more specific ordinary social roles and occupations, which refer to high accessibility and common prevalence. The “everyday” context also restricts the interpretation of heroism by concretizing it, which makes Everyday Hero more distinctive from other hero-related concepts, such as celebrities, sport stars, role models and martyrs [1,4,53,54].

Concerning the publicity dimension, several global hubs of Everyday Hero are occupations or ordinary roles that imply the lack of publicity, which is in line with Goethals and Allison’s [2] Transparent Hero type. The Hero network does not have global hubs expressing that publicity is a necessary characteristic.

The global hubs of Hero are too abstract to apply the range dimension. The ordinary social roles and occupations in Everyday Hero imply actions that have only a local effect. However, numerous roles and occupations are present and they often refer to Life-Long Heroism and Professional/”911” Heroism [8]. Therefore, it is important to consider their cumulative impact on society.

Hero and Everyday Hero cannot be differentiated on the dimension of challenge. Both have “self-sacrificing” as a global hub. None of them have other global hubs that define the magnitude of the physical risk or social sacrifice more precisely.

In sum, the abstract elements in the Hero network have a broad range of interpretations. Therefore, it is difficult to compare them to the concrete, social role and occupation-related connotations in the Everyday Hero network.

### Modular hubs in the Everyday Hero network

Several scholars suggested multiple categories for heroism [1,2,8]. Hence, we expected that Hero would have a modular structure that could be interpreted in accordance with prior categorizations. However, the Hero network is non-modular. Contrary to Hero, the social representation of Everyday Hero includes five modules: Pro-social Heroism, Prototypical Hero, Heroic Roles, Everyday Context and Ordinary Heroism (Fig 2). In the Hero network, a large proportion of the nodes express social roles, occupations or social contexts. However, they are not organized into modules. This means that the presence of similar elements does not guarantee that they will form coherent units in the structure of the social representation. Next, we describe the modules of the Everyday Hero network.

The modular hubs of Pro-social Heroism are “helpful”, “selfless”, “self-sacrificing”, “modest”, “kind”, “mindful” and “love” (Table 3). The hubs of this module vary in a broad spectrum from simply being kind to being ready for self-sacrifice. This module emphasizes the sociocentric mindset behind everyday heroism instead of an egocentric one [1]. Furthermore, this module shows high resemblance with the moral modeling function defined by Kinsella et al. [3].

The modular hubs of Prototypical Hero are “brave”, “endurance”, “honest” and “strong” (Table 3). This module contains internal, personality-related characteristics that are genuinely heroic. They refer to high competences or abilities [2]. This module overlaps to the largest extent with the social representation of Hero (see the details below). This module is in line with the enhancement function defined by Kinsella et al [3].

The modular hubs of Heroic Roles are “fireman”, “ambulance man”, “doctor”, “policeman” and “mother” (Table 3). The hubs of this module can be interpreted in the light of the protection function in the hero functions framework [3]. However, in this case the protection function is embedded in predefined social roles. On the basis of Franco et al. [1], these elements can be differentiated regarding the type of social sacrifice or physical risk.

The modular hubs of Everyday Context are “work”, “successful” and “family” (Table 3). This module can highlight the local contexts in which everyday heroism is exhibited. It seems that family and work might be strongly connected themes in the social representation of everyday heroism, which is in line with Goethals and Allison [2] and Allison and Goethals [53].

Ordinary Heroism does not have any modular hubs (Table 3). This facet of heroism can grasp the ordinary nature of heroic acts, which is related to accessibility, transparency and reward-independency revealed in previous research [1,2,8].

### Common associations in the Hero and Everyday Hero networks

Numerous common associations imply a similar meaning in social representations [16,17]. The common associations can reflect the most important heroic contents that do not change even if the context is altered. However, we can also use them to capture differences in social representations as the common associations can have different structural positions in the two social representations. The correlation of edge weights in the common association networks shows that there is indeed a difference in the patterns of connections.

Abric [15,17,18,19] argued that the central core elements are the stable parts of the social representations in altering environment. We found that most of the global hubs of both association networks are among the common associations. However, the global hubs of one association network do not necessarily have the same importance in the other one, which shows that a change in the context (like adding “everyday”) can significantly overwrite the hierarchy.

These findings point out the relevance of connections in understanding how the meaning emerges from the elements of the social representation. For example, we argue that bravery in connection with strength and self-sacrifice refers to a different connotation than bravery in connection with helpfulness, selflessness and self-sacrifice. While the first version implies a rescuer behavior, the other one implies a milder form of heroism such as standing up for someone in an offending situation.

### Limitations

In case of continuous associations, a couple of problems emerge such as retrieval inhibition or response chaining [56,57]. Retrieval inhibition happens when subsequent associations are produced with the same cue and retrieved information blocks new information. Response chaining happens when participants produce associations based on a previous response instead of the cue. It is not entirely presumable how strong the effect of a given response is on the next one. Association chaining and retrieval inhibition might influence the association process in individual cases but we hope that in a large dataset all significant layers of a given social representation eventually appear. Furthermore, chaining effect and retrieval inhibition can be reduced if the number of associated words is limited, which is the case in our study [56,57].

Previous studies demonstrated that the order of associations matters: first associations tend to be stronger than the next ones [56,57]. In the present study, we rejected the integration of the order into the network analysis, because we could not determine a non-arbitrary network weighting solution for the phenomenon. Therefore, we have considered all associations equal.

We supposed that our cues activated complete cognitive schemas. Hence, we considered a person’s associations as a coherent unit. It is also possible that a person’s associations are derived from different cognitive schemas. Nonetheless, a person’s associations are definitely connected by the fact that they are produced by the same person, so the construction of a network in itself is a valid step. However, the exact relations of a person’s associations are yet to be clarified.

It is also important to note that there are many algorithms for detecting modules in a network (for a review see [58]). It is also possible that there are algorithms that suit social representations better. For example, free association networks could be analyzed using overlapping modules [31].

### Future research

In Abric’s [15] central core vs. periphery hypothesis, the mean rank is also an important factor. The mean rank depends on the order of an individual’s associations. Several studies on associations argued that the order of associations matters [15,52,56,57,59]. The order is in connection with response chaining and retrieval inhibition as well. In the future, it would be interesting to develop a more refined edge weighting system that incorporates the order of associations.

Abric [15,17,18,19] argued that changes start from the periphery of the social representation and they gradually reach the central core. However, the propagation of information in complex networks is determined by the central nodes rather than the peripheral ones [60,61]. A future study could observe how new information is incorporated into social representations from a network perspective.

Free associations provide a limited insight into social representations. With this method, we do not have knowledge about the context of a given association, which means that we do not know how it relates exactly to the social representation. A solution to this problem could be to extract data with better consideration of the context using data-mining techniques [62]. For example, short texts can be processed and the key expressions can be transformed into a network. This provides more freedom for study participants to express themselves.

The sizes of the present datasets might not show the real power of network analysis. Algorithms can be employed efficiently even in case of millions of data points, which enables us to enlarge the data virtually without limits.

## Conclusion

In this study, we explored the social representations of Hero and Everyday Hero in Hungary. This research is a starting point of a long-term research project which aims to provide guidelines for motivating everyday heroic acts in communities. Everyday Hero has more concrete contents which are mainly present in Heroic Roles, Ordinary Heroism and Everyday Context modules. In case of both Hero and Everyday Hero, the abstract values and characteristics are in accordance with previous inductive studies [2,4,5,6]. Changing the topic from “hero” to “everyday hero” resulted in not only a different content but it created an entirely new network structure i.e., different global hubs (central cores), modular organization and more balanced connections of heroic contents. While heroism depicts doing something extraordinary in an abstract manner, everyday heroism implies just doing the right thing and it points out the ordinary roles, occupations and contexts in which the heroic values can be exhibited. Great heroes are truly inspirational. However, we suppose that encouraging individuals to behave heroically in their everyday lives can be more efficient if we approach heroism in a concrete and realistic manner.

## Supporting Information

S1 File Hero normalized node strengths. (XLS)

S2 File Everyday Hero normalized node strength. (XLS)

S3 File Everyday Hero normalized intra-modular node strength. (XLS)

S4 File Common associations. (XLS)

S5 File Minimal data set for Hero. This is the English translation of the raw data. We translated from Hungarian to English only those associations which occurred at least twice.
(XLS)

S6 File Minimal data set for Everyday Hero. This is the English translation of the raw data. We translated from Hungarian to English only those associations which occurred at least twice.
(XLS)

## References

[1] Franco Z E, Blau K, Zimbardo P G. Heroism: A conceptual analysis and differentiation between heroic action and altruism. Review of General Psychology. 2011;15(2):99–113.

[2] Goethals G R, Allison S T. Making Heroes: The Construction of Courage, Competence, and Virtue. Advances in Experimental Social Psychology. 2012;46:183–235.

[3] Kinsella E L, Ritchie T D, Igou E R. Lay perspectives on the social and psychological functions of heroes. Frontiers in Psychology. 2015;6. 10.3389/fpsyg.2015.00130 PMC4330705 25741302

[4] Kinsella E L, Ritchie T D, Igou E R. Zeroing in on heroes: A prototype analysis of hero features. Journal of Personality and Social Psychology. 2015;108(1):114–127. 10.1037/a0038463 25603370

[5] Rankin L E, Eagly A H. Is his heroism hailed and hers hidden? Women, men and the social construction of heroism. Psychology of Women Quarterly. 2008;32(4):414–422.

[6] Sullivan M P, Venter A. Defining Heroes Through Deductive and Inductive Investigations. The Journal of Social Psychology. 2010;150(5):471–484. 10.1080/00224540903366602 21058575

[7] Sullivan M P, Venter A. The Hero Within: Inclusion of Heroes into the Self. Self and Identity. 2005;4(2):101–111.

[8] Farley F. The Real Heroes of "The Dark Knight" [Internet]. Psychology today. 2012 [cited 17 June 2015]. Available from: https://www.psychologytoday.com/blog/the-peoples-professor/201207/the-real-heroes-the-dark-knight

[9] Smith S F, Lilienfeld S O, Coffey K, Dabbs J M. Are psychopaths and heroes twigs off the same branch? Evidence from college, community, and presidential samples. Journal of Research in Personality. 2013;47(5):634–646.

[10] Staats S, Hupp J, Wallace H, Gresley J. Heroes Don't Cheat: An Examination of Academic Dishonesty and Students' Views on Why Professors Don't Report Cheating. Ethics & Behavior. 2009;19(3):171–183.

[11] Davis J L, Burnette J L, Allison S T, Stone H. Against the odds: academic underdogs benefit from incremental theories. Social Psychology of Education. 2010;14(3):331–346.

[12] Zimbardo P G. The Lucifer effect. New York: Random House; 2007.

[13] Moscovici S. La psychanalyse, son image, son public. Paris: Presses Universitaires de France; 1961.

[14] Moscovici S. The phenomenon of social representations In: Moscovici S, ed. by. Social Representations. 1st ed Cambridge: Cambridge University Press; 2015 p. 210–289.

[15] Abric J C. Méthodologie de recueil des représentations sociales In: Abric J C, ed. by. Pratiques sociales et representations/Social Practices and Representations. 1st ed Paris: Presses Universitaire de France; 1994 p. 59–82.

[16] Kirchler E, Maciejovsky B, Schneider F. Everyday representations of tax avoidance, tax evasion, and tax flight: Do legal differences matter?. Journal of Economic Psychology. 2003;24(4):535–553.

[17] Abric J C. Coopération, compétition et représentations sociales. Cousset (Fribourg, Suisse): DelVal; 1987.

[18] Abric J C. ‘Les représentations sociales: Aspect théorique’. In: Abric J C, ed. by. Pratiques sociales et representations/Social Practices and Representations. 1st ed Paris: Presses Universitaire de France; 1994 p. 11–36.

[19] Abric J C. L’approche structurale des représentations sociales: développements récents. Psychologie et société. 2002;4(2):81–103.

[20] Érdi P, Makovi K, Somogyvári Z, Strandburg K, Tobochnik J, Volf P et al Prediction of emerging technologies based on analysis of the US patent citation network. Scientometrics. 2012;95(1):225–242.

[21] Böde C, Kovács I, Szalay M S, Palotai R, Kocsmáros T, Csermely P. Network analysis of protein dynamics. Febs Letters. 2007;581(15):2776–2782. 17531981 10.1016/j.febslet.2007.05.021

[22] Stam C V, Van Straaten E C W. The organization of physiological brain networks. Clinical Neurophysiology. 2012;123(6):1067–1087. 10.1016/j.clinph.2012.01.011 22356937

[23] Burt R S, Kilduff M, Tasselli S. Social Network Analysis: Foundations and Frontiers on Advantage. Annu Rev Psychol. 2013;64(1):527–547. 23282056 10.1146/annurev-psych-113011-143828

[24] Steyvers M, Tenenbaum J B. The Large-Scale Structure of Semantic Networks: Statistical Analyses and a Model of Semantic Growth. Cognitive Science. 2005;29(1):41–78. 10.1207/s15516709cog2901_3 21702767

[25] Guimerà R, Nunes Amaral L A. Functional cartography of complex metabolic networks. Nature. 2005;433(7028):895–900. 15729348 10.1038/nature03288 PMC2175124

[26] Knyazev G G, Volf N V, Belousova L V. Age-related differences in electroencephalogram connectivity and network topology. Neurobiology of Aging. 2015;36(5):1849–1859. 10.1016/j.neurobiolaging.2015.02.007 25766772

[27] Li L, Alderson D, Doyle J, Willinger W. Towards a Theory of Scale-Free Graphs: Definition, Properties, and Implications. Internet Mathematics. 2005;2(4):431–523.

[28] Meunier D, Achard S, Morcom A, Bullmore E. Age-related changes in modular organization of human brain functional networks. NeuroImage. 2009;44(3):715–723. 10.1016/j.neuroimage.2008.09.062 19027073

[29] González M C, Herrmann H J, Kertész J, Vicsek T. Community structure and ethnic preferences in school friendship networks. Physica A: Statistical Mechanics and its Applications. 2007;379(1):307–316.

[30] Jonsson P F, Bates P A. Global topological features of cancer proteins in the human interactome. Bioinformatics. 2006;22(18):2291–2297. 16844706 10.1093/bioinformatics/btl390 PMC1865486

[31] Palla G, Derényi I, Farkas I, Vicsek T. Uncovering the overlapping community structure of complex networks in nature and society. Nature. 2005;435(7043):814–818. 15944704 10.1038/nature03607

[32] Vergés P. Approche du noyau central: propriétés quantitatives et structurales In: Guimelli C, ed. by. Structures et transformations des representations sociales/Structure and Transformation of Social Representations. 1st ed Neuchâtel: Delachaux et Niestlé; 1994 p. 233–253.

[33] Spears N, Royne M, Van Steenburg E. Are Celebrity-Heroes Effective Endorsers? Exploring the Link between Hero, Celebrity, and Advertising Response. Journal of Promotion Management. 2013;19(1):17–37.

[34] Becker S W, Eagly A H. Comparing the Heroism of Women and Men. American Psychologist. 2005;60(4):343–344. 15943535 10.1037/0003-066X.60.4.343

[35] Johnson R C. Attributes of Carnage medalists performing acts of heroism and of the recipients of these acts. Ethology and Sociobiology. 1996;17(5):355–362.

[36] Lyons M T. Who are the Heroes? Characteristics of People Who Rescue Others. Journal of Cultural and Evolutionary Psychology. 2005;3(3):245–254.

[37] Elinder M, Erixson O. Every Man for Himself! Gender, Norms and Survival in Maritime Disasters. SSRN Journal. 10.1073/pnas.1207156109 PMC3421183 22847426

[38] Uhlmann E, Zhu L, Tannenbaum D. When it takes a bad person to do the right thing. Cognition. 2013;126(2):326–334. 10.1016/j.cognition.2012.10.005 23142037

[39] Williams T. Need-based heroism: the motivation to assign heroic status to others (2013). Honors Theses. Paper 6.

[40] Flament C, Rouquette M L. Anatomie des idées ordinaires. Paris: A. Colin; 2003.

[41] Karypis G. Evaluation of item-based top-n recommendation algorithms Proceedings of the tenth international conference on Information and knowledge management. ACM; 2001 p. 247–254.

[42] Billsus D, Pazzani M J. Learning Collaborative Information Filters. ICML 1st ed 2015 p. 46–54.

[43] Barabási AL, Albert R. Emergence of Scaling in Random Networks. Science. 1999;286(5439):509–512. 10521342 10.1126/science.286.5439.509

[44] Clauset A, Shalizi C, Newman M. Power-Law Distributions in Empirical Data. SIAM Rev. 2009;51(4):661–703.

[45] Newman M. Fast algorithm for detecting community structure in networks. Physical Review E. 2004;69(6). 10.1103/PhysRevE.69.066133 15244693

[46] Blondel V D, Guillaume J L, Lambiotte R, Lefebvre E. Fast unfolding of communities in large networks. J Stat Mech. 2008;2008(10):P10008.

[47] Sun Y, Danila B, Josić K, Bassler K E. Improved community structure detection using a modified fine-tuning strategy. Europhys Lett. 2009;86(2):28004.

[48] Rubinov M, Sporns O. Weight-conserving characterization of complex functional brain networks. NeuroImage. 2011;56(4):2068–2079. 10.1016/j.neuroimage.2011.03.069 21459148

[49] Nagarajan R, Kalinka A, Hogan W. Evidence of community structure in Biomedical Research Grant Collaborations. Journal of Biomedical Informatics. 2013;46(1):40–46. 10.1016/j.jbi.2012.08.002 22981843 PMC4121986

[50] Jacomy M, Venturini T, Heymann S, Bastian M. ForceAtlas2, a Continuous Graph Layout Algorithm for Handy Network Visualization Designed for the Gephi Software. PLoS ONE. 2014;9(6):e98679 10.1371/journal.pone.0098679 24914678 PMC4051631

[51] Albert R, Jeong H, Barabási AL. Error and attack tolerance of complex networks. Nature. 2000;406(6794):378–382. 10935628 10.1038/35019019

[52] Wachelke J. Representations and social knowledge: An integrative effort through a normative structural perspective. New Ideas in Psychology. 2012;30(2):259–269.

[53] Allison S T, Goethals G R. Heroes. Oxford: Oxford University Press; 2011.

[54] Bélanger J, Caouette J, Sharvit K, Dugas M. The psychology of martyrdom: Making the ultimate sacrifice in the name of a cause. Journal of Personality and Social Psychology. 2014;107(3):494–515. 10.1037/a0036855 25133728

[55] Wansink B, Payne C R, Van Ittersum K. Profiling the heroic leader: Empirical lessons from combat-decorated veterans of World War II. The Leadership Quarterly. 2008;19(5):547–555.

[56] De Deyne S, Storms G. Word associations: Norms for 1,424 Dutch words in a continuous task. Behav Res. 2008;40(1):198–205. 10.3758/brm.40.1.198 18411543

[57] Nelson D L, Mcevoy C L, Dennis S. What is free association and what does it measure?. Memory & Cognition. 2000;28(6):887–899. 11105515 10.3758/bf03209337

[58] Fortunato S. Community detection in graphs. Physics Reports. 2010;486(3–5):75–174.

[59] Wachelke J. Índice de centralidade de representações sociais a partir de evocações (INCEV): exemplo de aplicação no estudo da representação social sobre envelhecimento. Psicol Reflex Crit. 2009;22(1):102–110.

[60] Zeng A, Zhang C. Ranking spreaders by decomposing complex networks. Physics Letters A. 2013;377(14):1031–1035.

[61] Morales AJ, Borondo J, Losada JC, Benito RM. Efficiency of human activity on information spreading on Twitter. Social Networks. 2014 10 31;39:1–1.

[62] Wilkinson D M, Huberman B A. A method for finding communities of related genes. Proceedings of the National Academy of Sciences. 2004;101(Supplement 1):5241–5248. 10.1073/pnas.0307740100 PMC387302 14757821

